# Fatal large-vessel cerebrovascular infarct presenting with severe coronavirus disease 2019 in a 39-year-old patient: a case report

**DOI:** 10.1186/s13256-021-02991-3

**Published:** 2021-10-26

**Authors:** Nicolas Koslover, Marc Hardwick, Alexander Grundmann, Tamara Levene

**Affiliations:** 1 Barnet General Hospital, London, UK; 2 Brighton and Sussex University Hospital, Brighton, East Sussex UK; 3 Newcastle-Upon-Tyne Hospitals NHS Foundation Trust, Tyneside, UK; 4 Royal Free London NHS Foundation Trust, London, UK

**Keywords:** Anticoagulation, Covid-19, Stroke, Malignant stroke

## Abstract

**Background:**

Emerging reports are describing stroke in young, otherwise healthy patients with coronavirus disease 2019, consistent with the theory that some of the most serious complications of coronavirus disease 2019 are due to a systemic coagulopathy. However, the relevance of both the severity of coronavirus disease 2019 illness and established vascular risk factors in these younger patients is unknown, as reports are inconsistent.

**Case presentation:**

Here we describe a 39-year-old white male, who died after presenting simultaneously with a malignant large-vessel cerebrovascular infarct and a critical coronavirus disease 2019 respiratory illness. Doppler ultrasound revealed evidence of carotid plaque thrombosis. Blood tests revealed evidence of undiagnosed diabetes mellitus; however, the patient was otherwise healthy, fit, and active.

**Conclusions:**

This unique case highlights a possible interaction between established risk factors and large-vessel thrombosis in young patients with coronavirus disease 2019, and informs future research into the benefits of anticoagulation in these patients.

## Introduction

Current theories postulate that the most serious complications of coronavirus disease 2019 (COVID-19), including multiple reports of cerebrovascular disease [1], may be secondary to a systemic coagulopathy increasing the risk of thrombosis and the formation of microemboli [2]. This is thought to be a direct consequence of binding of the virus to angiotensin converting enzyme-2 (ACE2) receptors on the endothelial vessel lining [3], or the development of an antiphospholipid antibody [4]. A small but growing number of reports have now described stroke in younger COVID-19 patients [5–7]. However, conclusions surrounding the effect of established vascular risk factors in these cases are inconsistent. In addition, the relationship with the severity of COVID-19 disease is unclear, as only COVID-19 symptoms were mentioned in those patients not admitted to intensive care. The following case describes a unique scenario not previously reported, and highlights these interactions as crucial areas for further research.

## Emergency presentation

A 39-year-old white male developed sudden onset dysphasia while at home. An ambulance was called approximately 33 minutes following symptom onset, by which point he had begun to exhibit right-sided hemiplegia and facial weakness. The patient had been self-isolating at home for approximately 2 weeks owing to symptoms of cough, fever, shortness of breath, and vomiting.

He presented to the emergency department 2 hours and 24 minutes following the onset of symptoms. The patient was reported to have no past medical history and took no medications. Neurological examination confirmed right facial, leg, and arm weakness with associated right-sided sensory loss. He remained dysphasic and had evidence of dysarthria. NIHSS score was calculated as 13 and suggested a right partial anterior circulation stroke. Computed tomography (CT) head imaging 8 minutes after arrival was reported normal. Thrombolysis with recombinant tissue plasminogen activator (r-TPA) was initiated 56 minutes following arrival. A repeat NIHSS hours following thrombolysis was recorded as 18.

**Fig. 1:**
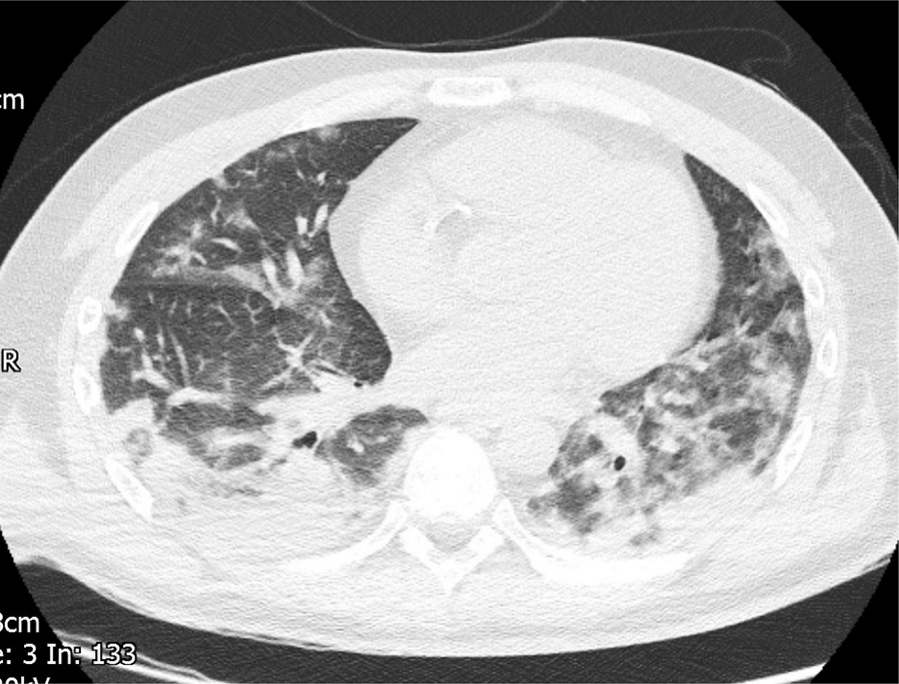
Computed tomography Thorax showing considerable covid pneumonitis

The patient was also tachypneic with respiratory distress and sinus tachycardia. Capillary blood glucose was measured as 27.6 mmol/L. Arterial blood gas analysis on 21% oxygen revealed type 1 respiratory failure with a partial pressure of oxygen (PaO2) of 5.85 kPa. He was started on 10 L/minute of oxygen. Chest X-ray revealed extensive bilateral peripheral predominant opacification involving upper mid and lower zones. High-resolution CT chest (Fig. 1) was formally reported to show moderately severe bilateral ground-glass changes affecting all lobes, consistent with COVID-19-related features. Nasopharyngeal aspirates for severe acute respiratory syndrome coronavirus 2 (SARS-CoV-2) were taken and later returned negative; however, in view of his previous symptoms and CT chest findings, he was deemed COVID-19 positive and isolated.

Blood tests revealed a C-reactive protein (CRP) of 150 mg/L, but full blood count, clotting studies, and thyroid, renal, and liver function all returned normal. Hemoglobin A1C (HbA1C) was raised at 109 nmol/mol, and a random lipid profile revealed raised triglycerides (5.24 mmol/L) and non-HDL cholesterol (3.58 mmol/L) along with reduced HDL cholesterol (0.92 mmol/L).

Initial therapy with empiric broad-spectrum antibiotics was initiated for possible aspiration pneumonia. Over the subsequent 48 hours, Glasgow Coma Score (GCS) remained between 12 and 14, and inhaled oxygen was reduced to maintain target oxygen saturation.

## Further clinical evaluation

**Fig. 2:**
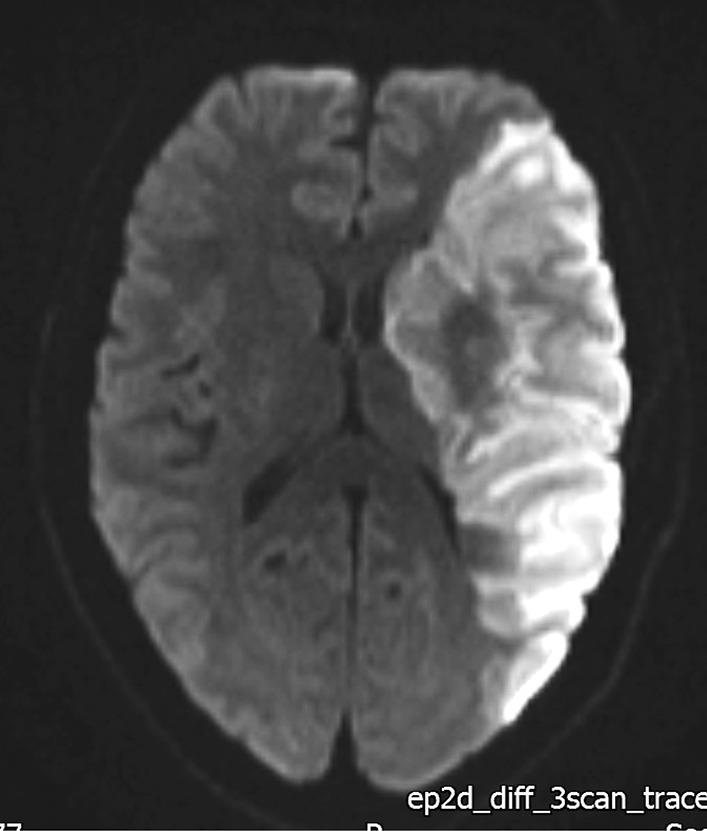
Diffusion-weighted MRI Brain showing restricted diffusion in the region of the left middle cerebral artery

An magnetic resonance imaging (MRI) scan (Fig. 2) performed the following day showed an extensive cerebral infarction in the left middle cerebral artery territory with no mass effect or midline shift.

Doppler ultrasound of the carotids revealed evidence of restricted flow in the left common carotid artery and thrombus along sections of the left internal carotid artery intracranially. Right-sided arteries were patent.

## Deterioration

**Fig. 3.**
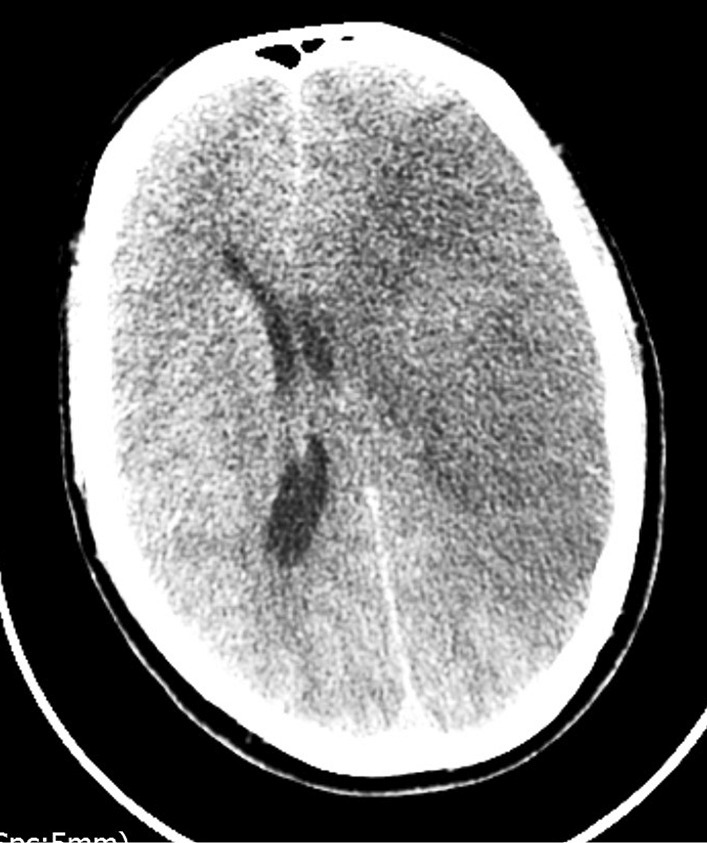
Computed tomography Brain showing L-sided oedema with mass effect

On the third day following admission, the patient underwent further combined neurological and respiratory deterioration. He required 15 L/minute oxygen to maintain target saturation and became febrile with temperature of 39 °C. He remained tachypneic, tachycardic, and normotensive. The GCS decreased to between 8 and 10, and the left pupil became fixed and dilated. Repeat CT scan was performed (Fig. 3) and revealed evidence of a dense left middle cerebral artery (MCA) ischemic infarct with mass effect and 1 cm midline shift to the right, resulting in hydrocephalus and raised intracranial pressure. Neurosurgical intervention was discussed but not considered feasible. The patient was managed conservatively and unfortunately died that evening.

## Discussion

In further support for the COVID-19 leading to a prothrombotic state, this patient’s COVID-19 related respiratory illness was contemporaneous with his cerebrovascular infarct and mirrored its severity, strongly suggesting a mechanistic relationship. This is consistent with some reports describing stroke in patients managed for COVID-19 in intensive care [8], but in most reports the temporal relationship is not clear.

The patient was apparently fit and healthy prior to his presentation; however, initial investigations revealed undiagnosed severe diabetes mellitus as well as a deranged lipid profile. Given his age and background, it is unclear whether these factors increased the risk of his large vessel thrombosis through a typical atherosclerotic mechanism. Although established atherosclerotic risk factors are considered important predictors for the severity of COVID-19 [9], available reports have not been able to consistently associate these with large-vessel thrombosis in younger COVID-19 patients [5–7].

Prospective studies are now needed to further characterize the associations between COVID-19 and cerebrovascular disease and other thrombotic disorders, as well as establish causatively whether and how vascular risk factors might influence the prothrombotic state independently from atherosclerosis. This will enable a crucial quantitative measurement of risk that can inform decisions regarding anticoagulation, which in such young patients with low bleeding risk is likely to be optimal management.

## Conclusion

This case adds to the small but growing body of reports describing young patients suffering cerebrovascular thromboembolic events during COVID-19 infection, including a limited number suffering large-vessel cerebral infarction [6]. As one of the youngest cases reported, and yet with clear evidence of diabetes mellitus, it illustrates a potential link between established vascular risk factors and the cerebrovascular complications of COVID-19 even in young patients. This case should prompt further investigation into the possible mechanisms for cerebrovascular complications of COVID-19 and how these may interact with the atherosclerotic state. This will help risk stratify patients into the optical treatment.

## Data Availability

The datasets during and/or analyzed during the current study are available from the corresponding author on reasonable request.

## References

[1] Markus HS, Brainin M. COVID-19 and stroke-a global world stroke organization perspective. Int J Stroke. 2020;15(4):361–4. 10.1177/1747493020923472 PMC11927026 32310017

[2] Wichmann D, Sperhake JP, Lütgehetmann M,* et al*. Autopsy findings and venous thromboembolism in patients with COVID-19. Ann Intern Med. 2020. 10.7326/L20-1206. 33316197

[3] Hess DC, Eldahshan W, Rutkowski E. COVID-19-related stroke. Transl Stroke Res. 2020;11(3):322–5. 10.1007/s12975-020-00818-9 PMC7202903 32378030

[4] Zhang Y, Xiao M,* et al*. Coagulopathy and antiphospholipid antibodies in patients with Covid-19. N Engl J Med. 2020;382:e38. 10.1056/NEJMc2007575 PMC7161262 32268022

[5] Beyrouti R, Adams ME, Benjamin L,* et al*. Characteristics of ischaemic stroke associated with COVID-19. J Neurol Neurosurg Psychiatry. 2020;91(8):889–91. 10.1136/jnnp-2020-323586 PMC7231545 32354768

[6] Oxley TJ, Mocco J, Majidi S,* et al*. Large-vessel stroke as a presenting feature of Covid-19 in the young. N Engl J Med. 2020;382(20):e60. 10.1056/NEJMc2009787 PMC7207073 32343504

[7] Sweid A, Hammoud B, Weinberg JH,* et al*. Letter: thrombotic neurovascular disease in COVID-19 patients. Neurosurgery. 2020. 10.1093/neuros/nyaa254. PMC7313768 32496534

[8] Klok FA, Kruip MJHA, van der Meer NJM,* et al*. Incidence of thrombotic complications in critically ill ICU patients with COVID-19. Thromb Res. 2020;191:145–7. 10.1016/j.thromres.2020.04.013 PMC7146714 32291094

[9] Jordan RE, Adab P, Cheng KK. Covid-19: risk factors for severe disease and death. BMJ. 2020. 10.1136/bmj.m1198. 32217618

